# Radiomics Based on Single-Phase CTA for Distinguishing Left Atrial Appendage Thrombus from Circulatory Stasis in Patients with Atrial Fibrillation before Ablation

**DOI:** 10.3390/diagnostics13152474

**Published:** 2023-07-25

**Authors:** Xue Li, Yuyan Cai, Xiaoyi Chen, Yue Ming, Wenzhang He, Jing Liu, Huaxia Pu, Xinyue Chen, Liqing Peng

**Affiliations:** 1 Department of Radiology, West China Hospital, Sichuan University, Chengdu 610041, China; lixue19970217@163.com (X.L.); drchenxiaoyi0204@163.com (X.C.); dr_hewenzhang@163.com (W.H.); dr_liujing@126.com (J.L.); phxia0409@126.com (H.P.); 2 Department of Cardiology, West China Hospital, Sichuan University, Chengdu 610041, China; 15881098907@163.com; 3 West China School of Medicine, Sichuan University, Chengdu 610041, China; star50551@outlook.com; 4 CT Collaboration, Siemens Healthineers, Chengdu 610041, China; xinyue.chen@siemens-healthineers.com

**Keywords:** radiomics, atrial fibrillation, left atrial appendage, thrombus, filling defect

## Abstract

Differentiation of left atrial appendage thrombus (LAAT) and left atrial appendage (LAA) circulatory stasis is difficult when based only on single-phase computed tomography angiography (CTA) in routine clinical practice. Radiomics provides a promising tool for their identification. We retrospectively enrolled 204 (training set: 144; test set: 60) atrial fibrillation patients before ablation, including 102 LAAT and 102 circulatory stasis patients. Radiomics software was used to segment whole LAA on single-phase CTA images and extract features. Models were built and compared via a multivariable logistic regression algorithm and area under of the receiver operating characteristic curves (AUCs), respectively. For the radiomics model, radiomics clinical model, radiomics radiological model, and combined model, the AUCs were 0.82, 0.86, 0.90, 0.93 and 0.82, 0.82, 0.84, 0.85 in the training set and the test set, respectively (*p* < 0.05). One clinical feature (rheumatic heart disease) and four radiological features (transverse diameter of left atrium, volume of left atrium, location of LAA, shape of LAA) were added to the combined model. The combined model exhibited excellent differential diagnostic performances between LAAT and circulatory stasis without increasing extra radiation exposure. The single-phase, CTA-based radiomics analysis shows potential as an effective tool for accurately detecting LAAT in patients with atrial fibrillation before ablation.

## 1. Introduction

Atrial fibrillation (AF), the most common tachyarrhythmia, has a lifetime risk of approximately 25% at the age of 40 and its prevalence increases with advancing age [1,2]. It was reported in 2008 that patients with AF incurred incremental healthcare costs of USD 26 billion [3]. Moreover, AF is related to increased risks of cardiovascular diseases, ischemic stroke, and all-cause mortality [4]. The most serious AF-related complication is ischemic stroke, primarily resulting from embolism of left atrial appendage thrombus (LAAT) [5]. Left atrial appendage (LAA), with its long and curved blind-end structure, is usually in a state of slow blood flow, circulatory stasis, and even thrombosis (further aggravation of circulatory stasis can form a thrombus) due to a decrease in LAA contractility and function when AF occurs [6]. LAA was reported as the source of an estimated 57% thrombi in rheumatic AF and 91% thrombi in nonrheumatic AF, respectively [5]. Transcatheter radiofrequency ablation is increasingly performed for AF patients who are drug-refractory or not eligible for medication. However, LAAT is an absolute contraindication [7]. Thus, accurate detection of LAAT is important for treatment planning and risk stratification.

Transesophageal echocardiography is the gold standard for detecting LAAT; however, as a semi-invasive technique requiring anesthesia, it is operator-dependent, uncomfortable, and can cause fatal complications such as gastrointestinal laceration or perforation [8,9]. Before routine clinical AF ablation, electrocardiogram-gated pulmonary venography is performed to assess the size of pulmonary vein ostium, the size of the left atrium, and the LAAT. But this is based on single-phase computed tomography angiography (CTA) with a reported poor positive predictive value [10]. This is because filling defects on single-phase CTA makes it difficult to differentiate between LAAT and circulatory stasis. A delayed scanning protocol is a reasonable alternative to transesophageal echocardiography, but it results in additional radiation exposure [10]. Hur et al. [11] reported that dual-energy computed tomography (CT) would be helpful for detecting LAAT, but the unavailability in most hospitals hinders its wide application. Recently, radiomics with machine learning algorithms has been used as a tool to convert invisible, qualitative, radiological image information into mineable, high-dimensional, quantitative, mathematical data for analysis, in the hope of improving diagnostic performance and conducting prognostic evaluation [12]. These quantitative data analyzed from biomedical images including intensity, shape, size or volume, and texture features may reflect underlying pathophysiology information (the basic concept of the radiomics process) different from conventional clinical and laboratory results [12]. As for radiomics based on cardiac CT images, it has been gradually reported to be valuable in the identification of high-risk atherosclerotic lesions, assessment of plaque microenvironments or vulnerability, detection of myocardial ischemia, and differentiation of cardiac mass [13]. Several radiomics studies involving thrombi were carried out to investigate abdominal aortic aneurysms’ growth status, predict aggressive type 2 endoleaks after endovascular aneurysm repair, and identify cardioembolic stroke before recanalization [14,15,16]. So, radiomics has shown great application prospects in many fields and we have speculated that radiomics based on single-phase CTA could identify this potential pathological change between LAAT and circulatory stasis. Previous studies have confirmed that radiomics based on single-phase CTA could be used for detecting LAAT from circulatory stasis [17,18]. But these studies are limited to a small sample size and valvular heart disease. In this study, we aimed to evaluate the diagnostic efficacy of radiomics based on single-phase CTA in the differentiation of LAAT from circulatory stasis in AF patients.

## 2. Materials and Methods

### 2.1. Patients

**Figure 1:**
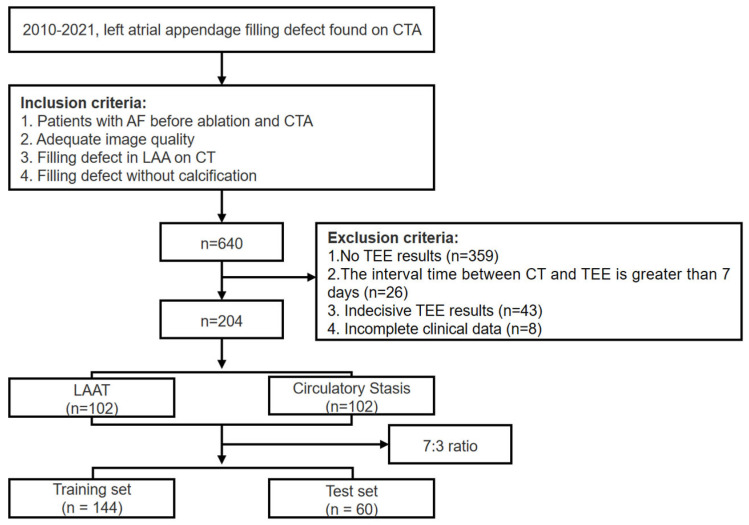
The patient recruitment workflow. CTA, computed tomography angiography; CT, computed tomography; TEE, transesophageal echocardiography; LAAT, left atrial appendage thrombus.

**Figure 2:**
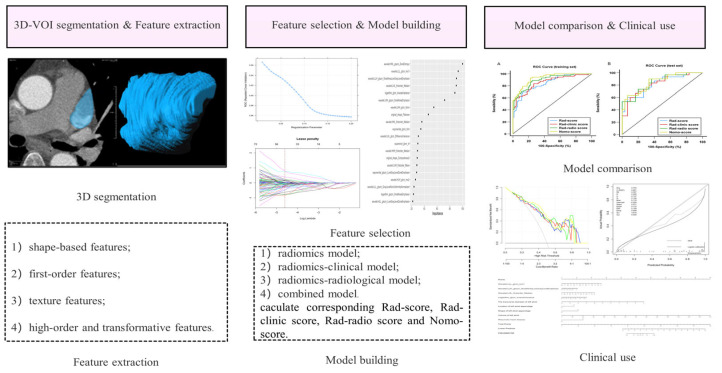
Radiomics workflow. 3D-VOI, 3-dimensional volume of interest.

The study was approved by the ethics committee of our institute. The informed consent was waived due to the retrospective nature of this study. A total of 640 patients with AF who were identified as having LAA filling defects via single-phase CTA in our hospital from January 2010 to August 2021 were retrospectively included. The inclusion criteria were as follows: (1) all subjects were hospitalized patients with AF before ablation and underwent routine preoperative CTA examination; (2) adequate image quality for analysis; (3) filling defect found on CTA images; (4) filling defect without calcification. The exclusion criteria were as follows: (1) absence of corresponding reference standard (transesophageal echocardiography results); (2) the interval time between CT and reference standard was greater than 7 days; (3) indecisive transesophageal echocardiography results; (4) incomplete clinical records. The patient recruitment workflow was shown in Figure 1. The clinical parameters were retrieved from the electronic medical records. The flowchart of this study was shown in Figure 2.

### 2.2. CT Image Acquisition and Analysis

The scanning range was from pulmonary trunk to the bottom of heart. All patients were injected with a total of about 50 mL of non-ionic iodinated contrast medium (400 mg I/mL) via the right median cubital vein at a flow rate of 5.0 mL/s via a power injector followed by 30 mL saline injection at the same rate. Subsequent enhanced scan with a retrospective electrocardiogram-gated acquisition was triggered by placing the region of interest in the descending aorta at a threshold of 200 Hounsfield units. CT images were reconstructed and stored in DICOM format. No β-blockers were used, and all patients were in the supine position.

The specific scanning parameters were shown in Table 1. Without knowing all patient identifiers and clinical information, two chest radiologists (with 5 and 15 years of experience, respectively) cooperated in collecting the radiological features. Any disagreement was solved by the final consensus. The assessed radiological features were as follows: CT attenuation value of filling defect, transverse diameter of LA (TD-_LA_), vertical diameter of LA (VD-_LA_), anteroposterior diameter of LA (AD-_LA_), volume of left atrium, position of LAA, and shape of LAA.

Using three-dimensional reconstruction technique, we measured the TD-_LA_ on the coronal plane, the VD-_LA_ and AD-_LA_ on the sagittal plane, while the LA volume was calculated according to the formula [Volume = 4/3π (TD-_LA_/2) (VD-_LA_/2) (AD-_LA_/2)] [19]. This study divided the shapes of LAA into chicken wing and non-chicken-wing according to previous literature [20]. The patient with ostium of LAA located between left superior pulmonary vein and left inferior pulmonary vein was recorded. Round region of interest of approximately 10 mm^2^ was drawn at the middle area of filling defect to calculate the CT attenuation value.

### 2.3. Transesophageal Echocardiography

The LAA filling defect was identified as thrombus or stasis (spontaneous echo contrast) via transesophageal echocardiography (iE33, Philips Healthcare, Best, The Netherlands). The LAA was assessed for thrombus or spontaneous echo contrast at the mid-esophageal level using 0-, 45-, 90-, and 135-degree views with Omni III 5 MHz probe. For a transesophageal echocardiography finding, LAAT was defined as a solid, well-circumscribed mass that was visible throughout the cardiac cycle, while spontaneous echo contrast was defined as dynamic, precipitous, viscid echo-density without a discrete mass [21]. Within the LAA, low-flow states were quantified via spectral Doppler. Each transesophageal echocardiography procedure was performed by one board-certified expert cardiologist with at least 5 years of experience, and the results were evaluated by two independent investigators. Any disagreement between them was solved by the final consensus.

### 2.4. VOI Segmentation for Radiomics

**Figure 3:**
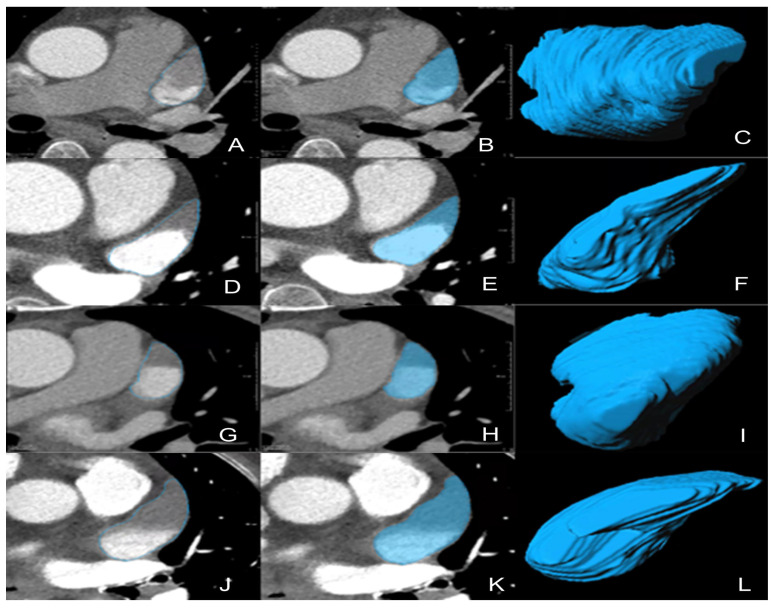
Representative 3D-VOIs segmentation. (**A**–**F**) LAA circulatory stasis; (**G**–**L**) LAAT; (**C**,**I**), non-chicken-wing-shaped LAA; (**F**,**L**), chicken-wing-shaped LAA. 3D-VOIs, 3-dimensional volumes of interest; LAA, left atrial appendage; LAAT, left atrial appendage thrombus.

For all the CTA images of the enrolled patients exported in DICOM format on the PACS, a radiologist performed manual segmentation of the whole LAA, slice-by-slice, on prototype software called “Radiomics” (Syngo. via Frontier, Vision 1.0.0, Siemens, Munich, Germany) to obtain three-dimensional volume of interest, as shown in Figure 3 [22]. The segmentation details were as follows: firstly, we located and marked the ostium of the LAA on the oblique coronal plane after multiplanar reconstruction. (For more details on evaluating the ostium, please refer to the article by Kasper et al. [23].) Subsequently, returning to the standard cross-sectional view, we noted the existence of the marked boundary between LAA and left atrium. Then, manual segmentation was performed along the clear margin of LAA, avoiding adjacent vessels and surrounding fat. Lastly, the top and bottom layers of the LAA were excluded to avoid the interference of partial volume effect.

### 2.5. Extraction and Selection of Radiomics Features

Our “Radiomics” prototype software (Syngo. via Frontier, Vision 1.0.0, Siemens, Germany) is a commercialized imaging research workstation of Siemens company, which is used to carry out generic radiomics research studies for clinicians. The software has both segmentation and radiomics feature extraction functions. Its radiomic feature computation relies on the de facto standard library, PyRadiomics (prototype interfaces with the PyRadiomics library in a similar manner to 3D Slicer’s Radiomics plugin) [22]. So, after segmentation on “radiomics”, the radiomics features were automatically extracted by using the PyRadiomics package (version 3.0, https://pyradiomics.readthedocs.io/en/latest accessed on 15 June 2023) [24]. Information on the three-dimensional volumes of interest was resampled to the equivalent points of voxel set in a normalized 1 × 1 × 1 mm^3^ template space to create an isotropic dataset (reducing the impact of pixel size and thickness) that were reproducible and comparable before feature calculations. Through “bin the feature,” “radiomics” automatically transformed the grayscale of the image into discrete integer values, which were recognizable by a computer. Then, “Radiomics” proceeded to the extraction process where different filters and image transformation methods were employed in the resampled volumes of interest. Except for shape features, which are intensity-independent and therefore unfiltered, first- and higher-order features were also calculated from the processed images via the filters available on the software platform. These filters include wavelet of different decomposition levels (levels: LLL, LLH, LHL, LHH, HLL, HLH, HHH, HHL), log, exponent, square, and square root functions. The radiomics features computation in our study is based on the plug-in from Pyradiamics [22]. Finally, four categories of radiomics features with 1226 were extracted from the original and post-processed three-dimensional volumes of interest: (1) 17 shape-based features, (2) 18 first-order features, (3) 65 texture features, (4) 1116 high-order and transformative features. 

To ensure the reliability of radiomics features, the regions of interest were re-segmented in 20 random instances (10 LAAT and 10 stasis) by another radiologist for inter-observer reproducibility analysis. Only the radiomics features with intraclass correlation coefficient greater than 0.75 were considered as having good reliability and selected for subsequent analysis [25]. The least absolute shrinkage and selection operator method, with penalty parameter tuning conducted with 5 repeats and 10-fold cross-validation, was used for feature selection. The detailed feature selection method was as follows. We first randomly allocated all enrolled patients to training set (*n* = 144) and test set (*n* = 60) at a ratio of 7:3 with the stratified random sampling technique. Then, we proceeded to train the model in the training set with 5 repeats and 10-fold cross-validation (50 resamples in total) using the least absolute shrinkage and selection operator regression. With tuning penalty parameter λ between 0.01–0.20, irrelevant variables were compressed, and those features with nonzero coefficients would be selected. The optimal λ was identified when the model achieved the highest average area under of the receiver operating characteristic curve on 50 resamples. We recorded this performance as optimal setting and derived the feature importance ranking list. Finally, to construct simplified radiomics model, appropriate number of features were selected based on the acceptable performance relative to the optimal setting. 

The statistically significant clinical and radiological features (corrected *p* < 0.05) with our aim of study in univariate regression were entered into the multivariate regression to construct the radiomics clinical model and radiomics radiological model, respectively. Same step was taken to establish the combined model by integrating clinical, radiological, and radiomics features. During the above modeling process, the features with insignificant correlation in the multiple regression model were deleted to ease the redundancy of the model.

### 2.6. Model Development and Evaluation

Based on the selected radiomics, clinical, and radiological features, 4 models were constructed: (1) the radiomics model using only radiomics features, (2) the radiomics clinical model using both radiomics and clinical features, (3) the radiomics radiological model using both radiomics and radiological features, and (4) the combined model integrating the radiomics, clinical and radiological features. 

The performance of all four prediction models was compared via the area under of the receiver operating characteristic curve (AUC). Differences in the AUC values between different models were compared using DeLong’s test. A calibration curve plotted graphically was used to evaluate the actual LAAT probability against the predicted of LAAT probability. Decision curve analysis was a tool to assess the clinical utility by calculating the net benefits at different threshold probabilities.

### 2.7. Statistical Analysis 

R software (version 1.1.453), MedCalc^®^ Statistical Software version 20 (MedCalc Software Ltd., Ostend, Belgium; https://www.medcalc.org (accessed on 10 May 2021)) and SPSS (version 26, SPSS Chicago, IL, USA) were used for performing all statistical descriptive analyses, with a two-tailed *p* < 0.05 being considered statistically significant. Shapiro–Wilk test, visual histogram, and Q-Q chart were used to check the normality of continuous variables. Continuous variables were assessed by using independent sample *t*-test (for those with normal distribution) and Mann–Whitney U test (for those with non-normal distribution), whereas categorical variables were calculated via the chi-square or Fisher exact tests. Normally and non-normally distributed data were expressed as mean ± standard deviation and median (P_25_–P_75_), respectively, whereas categorical variables were depicted as frequencies. Odds ratios (OR) with 95% confidence intervals (CI) were output as results of multiple regression analysis. 

## 3. Results

### 3.1. Clinical and Radiological Features

In this study, 102 cases with LAAT (mean age: 63.50 ± 11.07 years, range: 33–89 years) and 102 cases with LAA circulatory stasis (mean age: 68.70 ± 9.43 years, range: 40–89 years) were retrospectively enrolled, and the 204 cases were randomly assigned to training set (*n* = 144, 72 were LAAT) and test set (*n* = 60, 30 were LAAT). The mean time between standard references and CT scans was 1.72 ± 1.94 days (range: 0–7 days). Detailed comparisons of clinical and radiological features were summarized in Table 2 and Table 3. All parameters between the training and test sets showed no significant intra-group difference (all *p* > 0.05). The clinical characteristics between the LAAT group and stasis group were significantly different except for age, prothrombin time, international normalized ratio, activated partial thromboplastin time and thrombin time (all *p* > 0.05). In terms of radiological characteristics, the LAAT group had lower CT attenuation value, increased TD-_LA_, increased VD-_LA_, increased AD-_LA_, increased volume, more-chicken-wing-shaped LAAs, more LAAs located between left superior pulmonary vein and left inferior pulmonary vein (all *p* < 0.05) than the stasis group.

Univariate regression analysis using a Bonferroni correction demonstrated that NYHA (≥3/<3), rheumatoid heart disease (RHD) (±), CT value, TD-_LA_, VD-_LA_, AD-_LA_, volume, location (±) and shape (±) were associated with an increased risk of LAAT (all corrected *p* < 0.05). These features were then included in the stepwise multivariate analysis. At the multivariable analysis, the presence of RHD, increased TD-_LA_, increased volume of left atrium, LAA located between left superior pulmonary vein and left inferior pulmonary vein, and chicken-wing-shaped LAAs were screened out to be significantly predictive for the presence of LAAT [OR = 37.80, 0.36, 1.02, 7.90, and 11.80; *p* < 0.001, =0.024, =0.002, <0.001, and <0.001]. More comprehensive information of the univariate and the multivariate analysis was shown in Table 4.

### 3.2. Radiomics Analysis and Model Development

In this study, a total of 693 radiomics features with an inter-class correlation coefficient > 0.75 were entered into least absolute shrinkage and selection operator regression. The best tuned regularization parameter λ (λ = 0.01) we finally chose occurring when the model achieved the highest average AUC, corresponded to an optimal subset of four features for constructing the radiomics model. The specific features and their respective coefficients were shown in Table 5. The radiomics–clinical model was constructed by integrating RHD with radiomics features and radiomics–radiologic model was built by integrating TD-_LA_, volume, location, shape with radiomics features, respectively. Finally, we developed the combined model which was composed of three radiomics features (B, C, D, E), one clinical feature (RHD), and four radiological features (TD-_LA_, volume, location, shape). During the modeling process, to reduce feature redundancy in multivariate logistic regression model, variable A was removed from radiomics features for establishing the radiomics–clinical model, radiomics–radiologic model, and combined model, respectively. 

Radiomics scores based on four models were calculated from linear combinations of features weighted by their respective coefficients as follows: Rad-score based on radiomics model = 0.3003 − (0.2842 × A) + (1.3021 × B) − (0.4753 × C) + (0.5513 × D) − (1.1622 × E); Rad-clinic score based on radiomics–clinical model = −0.0389 + (1.1738 × B) − (0.2570 × C) + (0.6109 × D) − (1.0678 × E) + (3.2506 × RHD); Rad-radio score based on radiomics–radiological model = 5.9174 + (1.0871 × B) − (0.4312 × C) + (0.8865 × D) − (0.7409 × E) + (0.0220 × Volume) + (2.1819 × Location) + (2.8035 × Shape) − (1.4159 × TD-_LA_); Nomo-score based on combined model = 6.8991 + (1.1407 × B) − (0.4924 × C) + (1.0289 × D) − (0.8492 × E) + (0.0200 × Volume) + (2.3051 × Location) + (3.0992 × Shape) − (1.5481 × TD-_LA_) + (4.0192 × RHD).

### 3.3. Model Comparison

**Figure 4:**
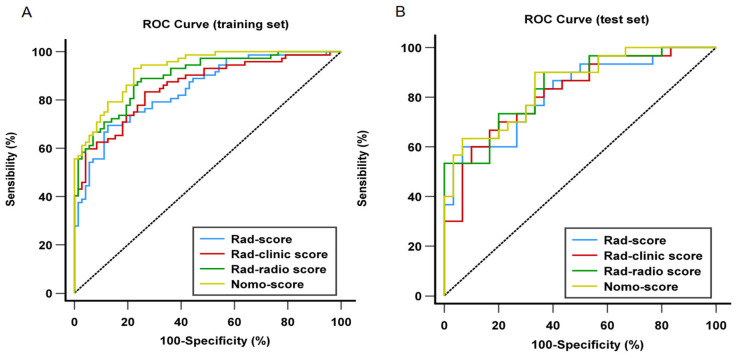
Comparison of receiver operating characteristic (ROC) curves of all four prediction models for distinguishing left atrial appendage thrombus from circulatory stasis in the training (**A**) and test (**B**) set. (**A**) In the training set, AUC = 0.93 for the Nomo-score; 0.90 for the Rad-radio score. 0.86 for the Rad-clinic score; and 0.82 for the Rad-score; (**B**) in the test set. AUC = 0.85 for the Nomo-score; 0.84 for the Rad-radio score; 0.82 for the Rad-clinic score; and 0.82 for the Rad-score. ROC, receiver operating characteristic curve; AUC, under of the receiver operating characteristic curve.

The ROC results of four models in the training and test sets are shown in Table 6 and Figure 4. The Rad-score achieved a discriminatory capacity with an AUC of 0.82 (95%CI: 0.75–0.89) in the training set and good performance was confirmed in the test set with an AUC of 0.82 (95%CI: 0.72–0.93). When adding RHD with radiomics features, the Rad-clinic score showed slightly better results than the Rad-score in the training set, which had an AUC of 0.86 (95%CI: 0.80–0.92) and was nearly equal in the test set, with an AUC of 0.82 (95%CI: 0.71–0.93). Improved predictive capacity was achieved in the Rad-radio score with an AUC of 0.90 (95%CI: 0.85–0.95) in training set and an AUC of 0.84 (95%CI: 0.75–0.94) in test set. Finally, of 4 models, the Nomo-score yielded the highest performance with an AUC of 0.93 (95%CI: 0.89–0.97) and an AUC of 0.85 (95%CI: 0.76–0.95) in the training and test sets, respectively. Delong test was performed on both training and test sets, and the results showed that there were significant differences between the Nomo-score and the Rad-score, the Nomo-score and the Rad-clinic score, the Rad-score and the Rad-radio score, the Rad-clinic score and the Rad-radio score in distinguishing LAAT from circulatory stasis in the training set (all *p* < 0.05). There were no significant differences found in the comparison of other models on both training and test sets (all *p* > 0.05) (Table 7).

**Figure 5:**
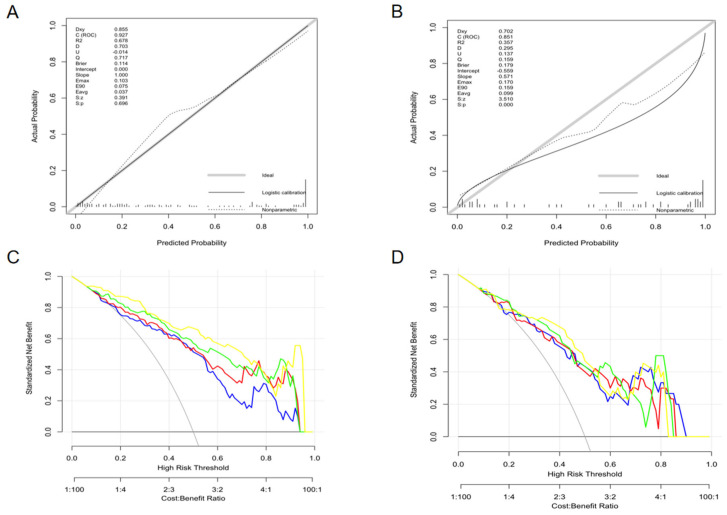
The calibration curves (training set: **A**; test set: **B**) and decision curves (training set: **C**; test set: **D**) of the combined nomogram. The calibration curves (**A**,**B**) depict the agreement of the combined model between the predicted risk and actual probability. The 45° gray line represents the ideal prediction, and the solid black line represents the actual probability of the model. The closer the solid line is to the ideal line, the better the predictive performance of the nomogram is. The decision curve (**C**,**D**) is used to assess the clinical utility by calculating the net benefits at different threshold probabilities. The x axis corresponds to the threshold probability while the y axis corresponds to the net benefit. The blue line, red line, green line, and yellow line represents net benefit of the Rad-score, Rad-clinic score, Rad-radio score, and Nomo-score, respectively. The grey line is made with the assumption that all patients are with LAAT. The black line is made with the assumption that no patients are with LAAT. (**C**) shows that the net benefit of the Nomo-score is better than the other cases when the threshold is not within the range of 78–91% in the training set. (**D**) Shows that if the threshold probability is less than 82%, the Nomo-score and Rad-radio score demonstrated more benefit than “LAAT all”, “Stasis all”, Rad-score, and the Rad-clinic score at almost all threshold probabilities in the test set.

The calibration curve of the nomogram (Figure 5A,B) illustrated good consistency between the predicted and actual probabilities of distinguishing LAAT from circulatory stasis. The decision curve is used to assess the clinical utility by calculating the net benefits at different threshold probabilities. It showed that the net benefit of the Nomo-score is better than the other cases when the threshold is not within the range of 78–91% in the training set (Figure 5C). Figure 5D shows that if the threshold probability is less than 82%, the Nomo-score and Rad-radio score demonstrated more benefits than “LAAT all”, “Stasis all”, Rad-score, and the Rad-clinic score at almost all threshold probabilities in the test set.

## 4. Discussion

LAA has become an attractive research hotspot as an important, treatable source of cardiovascular embolic stroke [8]. A review demonstrated that single-phase CTA had a promising negative predictive value of 99% for distinguishing LAAT from circulatory stasis, whereas its mean positive predictive value was only 41% [10]. Emerging CT modalities have been proposed to increase the diagnostic performance. Dual-phase CT using a double-injection scan protocol is viewed as a reliable alternative to transesophageal echocardiography, with its positive predictive value increased to 92%, while dual-enhanced CT implementing a double-contrast agent scan protocol could achieve a positive predictive value of 100% [10,26]. However, they can result in additional radiation exposure or increased contrast agents, even contrast-induced nephropathy. Hur et al. [11] reported that dual-energy CT based on rapidly switching tube voltage would be helpful for detecting LAAT, but the unavailability in most hospitals hinders its wide application. Compared with routine (supine position) CT, prone position CT is a promising tool for assessing intracardiac thrombi, but early prone position CT is still a challenge in excluding all thrombi from the filling defect [27]. For cardiac magnetic resonance (CMR), Kitkungvan et al. [28] showed that the delayed enhancement with a long-inversion-time CMR, less subject to the impact of artifact, had a superior accuracy (99.2%) to other CMR components. However, its time-consuming and costly nature is its biggest drawback.

In addition to the above qualitative methods, there are some quantitative methods such as calculating the LAA/ascending aorta (AA), the Hounsfield unit ratio, controlling the delay time of delayed scanning, and assessing the enhancement pattern based on dual-phase CT [17,29,30]. It is worth mentioning that, referring to the LAA/AA ratio, Chun et al. [17] confirmed that single-phase, CTA-based radiomics was superior to the early LAA/AA ratios and similar to the delayed LAA/AA ratios for identifying LAAT. Recently, previous studies based on single-phase CTA radiomics have shown potential in distinguishing LAAT from circulatory stasis [17,18]. But these studies were limited to a small sample size and valvular heart disease. 

Our 204 enrolled patients included a larger sample size, with a 1:1 match (LAAT group: circulatory stasis group = 102:102), than previous studies Chun et al. (25:70); Ebrahimian et al. (44:33), reducing selection bias and ensuring that our results possessed sufficient representativeness [17,18]. Previous studies have not incorporated inter-observer analysis, and the reproducibility of features may be open to doubt, while our inter-class correlation coefficient results show good reliability between radiologists [17,18]. In line with Ebrahimian et al. [18], considering the difficulty of delineating the ill-defined filling defect, we took the whole LAA as the volume of interest and achieved higher performance (AUC = 0.82) than Chun et al. [17], who used the filling defect as the volume of interest (AUC = 0.78), slightly inferior to Ebrahimian et al. [18] (AUC = 0.85). The higher performance than Chun et al. [17] may be explained by our lager sample size and manual segmentation method being different from their semi-automatic method. The slightly inferior performance to Ebrahimian et al. [18] may be due to different scanners, population heterogeneity, and different reference standards. Additionally, late-phase CT rather than gold-standard transesophageal echocardiography was used as the standard of reference in their study [18].

In our study, the first-order feature (Variable D), gray-level co-occurrence matrix features (Variable B, E) and gray-level size zone matrix features (Variable A, C) provided greater contributions to the radiomics model for distinguishing LAAT from circulatory stasis. These features consider number, distance, angle, etc. (refer to https://pyradiomics.readthedocs.io/en/latest/index.html accessed on 15 June 2023). Wavelet of different decomposition levels and logarithm were filters that used to help emphasize different aspect of the underlying image for radiomic analysis. Variable D means median gray level intensity within the region of interest. Variable C measures the proportion in the image of the joint distribution of smaller size zones with lower gray-level values. Variable A, Variable B, and Variable E measure the uncertainty/randomness, and the complexity and homogeneity of the texture (https://pyradiomics.readthedocs.io/en/latest/index.html accessed on 15 June 2023). Higher values in Variables A and B, and lower value in Variable E indicate more heterogeneneity in the texture patterns. In our study, higher variable A and B values and lower E value were found in the LAAT group, which may explain the potential pathological change that further aggravation of circulatory stasis forms thrombus.

Clinical and radiological information was easy to obtain, and the information we collected was meaningful in detecting LAAT. Chun et al. [17] compared radiomics results with the early and delayed LAA/AA ratio without building any combined models. Although a combined model was constructed in the study of Ebrahimian et al. [18] with an AUC = 0.92 basically identical to ours, it was only based on the integration of subjective radiologist assessment with radiomics features and did not include more reliable objective indicators than our study. With RHD (+), TD-_LA_, volume, shape (−), and location (+) being added, the combined model showed excellent performance (AUC = 0.93) in distinguishing LAAT from circulatory stasis, with robust results in the test set (AUC = 0.85). It was reported that the global prevalence of AF in RHD patients was 32.8%, and that AF patients with RHD have a 17-fold increased risk of stroke compared with an only 5-fold risk in AF patients alone [31,32]. Chicken-wing-shaped LAA appears to be a protective factor against LAAT, with the highest LAA ejection velocity compared to other morphologies [33]. The increased left atrium volume added in our model revealed a higher risk of LAAT, consistent with previous studies [34]. Fang et al. [35] reported that the lowest contractibility was found when the ostia of a LAA located between the left superior pulmonary veins and the left inferior pulmonary veins, rather than adjacent to the left superior pulmonary veins or the left inferior pulmonary veins, indicating a higher risk of LAAT.

There were several limitations in our study. Firstly, all samples were retrospectively collected from a single center and lacked external validation. A comprehensive and prospective multicenter survey is still needed. Secondly, we did not perform reference standards and corresponding CT scans on the same day, but the mean interval between them was 1.72 ± 1.94 days. Last but most important, the CT scanning parameters and reconstruction protocol (i.e., slice thickness and the field of view and reconstruction kernels) can influence the results of radiomics model features. In our study, image standardization was not performed when scanning and reconstructing images. However, this is unavoidable in clinical practice as different scanners are installed in an institution. This is the reason that, before feature calculation, we used different image pre-processing methods on the segmented volume, to emphasize the edges, smooth (overview emphasis), or sharpen (detail emphasis) the image with different degrees. Although standardizing scanning protocols is difficult to achieve, our combined model based on single-phase CTA exhibited an excellent performance for differentiating LAAT from circulatory stasis, which could help to provide more accurate diagnostic information to clinicians and avoid unnecessary radiation exposure and use of contrast agents.

## 5. Conclusions

The combined model exhibits excellent differential diagnostic performances between LAAT and LAA circulatory stasis without increasing radiation exposure. The single-phase, CTA-based radiomics analysis shows potentiality as an effective tool for accurately detecting LAAT in patients with AF, before ablation.

## Figures and Tables

**Table 1:** Computed tomography scanning parameters.

Manufacturer	SIEMENS (Munich, Germany), *n* = 120; GE, *n* = 71; UIH, *n* = 13
Image Extent (pixels)	512 × 512
Voxel Spacing (mm)	Mean ± SD: 0.640 ± 0.125; Median (P_25_–P_75_): 0.625 (0.625–0.700)
Slice Thickness (mm)	0.5, *n* = 4; 0.6, *n* = 3; 0.625, *n* = 71; 0.75, *n* = 38; 1.0, *n* = 88
Reconstruction diameter (mm)	Mean ± SD: 243.7 ± 48.9; Median (P_25_–P_75_): 231 (215–264)
Reconstruction Kernel	STANDARD, *n* = 90; I26f, *n* = 64; B26f, *n* = 23; DETALL, *n* = 14; C_SOFT_AA, *n* = 13
Tube voltage (kV)	80, *n* = 18; 100, *n* = 124; 120, *n* = 62
Tube Current (mA)	Mean ± SD: 887.8 ± 368.5; (P_25_–P_75_): 793 (582.5–1283)

Abbreviations: SD, standard deviation.

**Table 2:** Clinical characteristics in the training and test set.

Characteristics	LAAT Group (*n* = 102)	Stasis Group (*n* = 102)	*p* Value	Training Set (*n* = 144)	Test Set (*n* = 60)	*p* Value
Age (years)	63.50 ± 11.07	68.74 ± 9.43	<0.001	66.17 ± 11.09	65.98 ± 9.36	0.907
Sex (male/female)	48/54	57/45	0.207	70/74	35/25	0.206
NYHA (≥3/<3)	37/65	10/92	<0.001	33/111	14/46	0.949
CRI (±)	10/92	1/101	0.005	9/135	2/58	0.514
RHD (±)	34/68	3/99	<0.001	26/118	11/49	0.963
SUC (umol/L)	400.07 ± 128.43	363.61 ± 83.83	0.017	381.57 ± 108.73	382.49 ± 112.96	0.957
SC (±)	14/88	5/97	0.048	14/130	4/56	0.483
Albumin (g/L)	41.14 ± 4.15	42.20 ± 2.92	0.037	41.68 ± 3.86	41.65 ± 3.01	0.961
PT (s)	13.15	13.00	0.332	13.05	12.80	0.304
	(11.80–18.03)	(11.78–16.48)		(11.80–18.08)	(11.63–15.63)	
INR	1.15	1.11	0.242	1.14	1.11	0.122
	(1.04–1.60)	(1.03–1.42)		(1.04–1.56)	(1.01–1.41)	
APTT (s)	30.00	30.95	0.307	30.50	30.35	0.627
	(26.88–35.20)	(27.98–35.63)		(27.23–35.60)	(26.90–35.35)	
TT (s)	19.05	19.40	0.057	19.45	19.10	0.529
	(18.28–20.20)	(18.60–20.73)		(18.43–20.60)	(18.40–20.65)	
Fibrinogen (g/L)	3.06 ± 0.78	2.82 ± 0.62	0.016	2.98 ± 0.74	2.84 ± 0.64	0.215
WBC (10^9^/L)	6.70 ± 2.25	5.92 ± 1.59	0.005	6.17 ± 1.79	6.64 ± 2.37	0.120
RDW_sd_ (fL)	48.04 ± 4.73	46.62 ± 4.39	0.028	47.45 ± 4.64	47.04 ± 4.55	0.561
RDW_cd_ (%)	14.34 ± 2.13	13.84 ± 1.34	0.042	14.19 ± 1.96	13.85 ± 1.27	0.218

Abbreviations: NYHA, New York Heart Association functional class; CRI, chronic renal insufficiency; RHD, rheumatic heart disease; SUC, serum uric acid; SC (±), increased/normal serum creatinine; PT, prothrombin time; INR, international normalized ratio; APTT, activated partial thromboplastin time; TT, thrombin time; WBC, white blood cell; RDW_sd_, standard deviation of red blood cell volume distribution width; RDW_cd_, coefficient of variation of red blood cell volume distribution width.

**Table 3:** Radiological characteristics in the training and test set.

Characteristics	LAAT Group (*n* = 102)	Stasis Group (*n* = 102)	*p* Value	Training Set (*n* = 144)	Test Set (*n* = 60)	*p* Value
CT value (Hu)	54.00	78.50	<0.001	65.50	65.00	0.653
	(41.75–86.25)	(54.75–126.00)		(42.50–116.00)	(52.00–96.75)	
TD-_LA_ (cm)	8.61 ± 1.47	8.06 ± 0.93	0.002	8.38 ± 1.30	8.23 ± 1.14	0.461
VD-_LA_ (cm)	7.74 ± 1.36	7.22 ± 0.89	0.002	7.46 ± 1.18	7.52 ± 1.17	0.749
AD-_LA_ (cm)	5.32 ± 1.25	4.86 ± 0.78	0.002	5.11 ± 1.13	5.06 ± 0.91	0.770
Volume (cm^3^)	172.19	144.57	0.001	153.58	156.33	0.656
	(131.33–214.69)	(120.27–181.35)		(125.60–198.24)	(120.74–185.88)	
Position (±)	48/54	13/89	<0.001	41/103	20/40	0.490
Shape (±)	51/51	9/93	<0.001	39/105	21/39	0.258

Abbreviations: CT value indicates round region of interest of approximately 10 mm^2^ was drawn at the middle area of filling defect to calculate the CT value; Hu, Hounsfield unit; TD-_LA_, transverse diameter of left atrium; VD-_LA_, vertical diameter of left atrium; AD-_LA_, anteroposterior diameter of left atrium; Volume, volume of left atrium was calculated according to the formula [Volume = 4/3π (TD-_LA_/2) (VD-_LA_/2) (AD-_LA_/2)]; Position (±), ostium of LAA was located between left superior pulmonary vein and left inferior pulmonary vein/adjacent to left superior pulmonary vein or left inferior pulmonary vein; and Shape (±), chicken-wing-shaped LAA/non chicken-wing-shaped LAA.

**Table 4:** Univariable and multivariable logistic regression of LAAT.

Characteristics	Univariable				Multivariable		
	OR	95%CI	*p* Value	Corrected-*p*	OR	95%CI	*p* Value
NYHA	4.26	1.83–10.90	0.001	0.019			
CFI	8.87	1.57–167.00	0.042	0.798			
RHD	37.80	7.60–686.00	<0.001	0.019	37.80	4.95–288.07	<0.001
BUC	1.00	1.00–1.01	0.033	0.627			
SC	4.15	1.23–19.00	0.035	0.665			
Albumin	0.90	0.82–0.99	0.030	0.570			
PT	1.05	0.99–1.12	0.140	1			
INR	1.79	0.92–3.75	0.100	1			
APTT	1.01	0.97–1.06	0.600	1			
TT	0.99	0.98–1.00	0.200	1			
Fibrinogen	1.80	1.12–2.99	0.019	0.361			
WBC	1.27	1.04–1.58	0.022	0.418			
RDW_sd_	1.11	1.02–1.21	0.016	0.304			
RDW_cv_	1.22	0.99–1.56	0.093	1			
CT value	0.99	0.98–0.99	<0.001	0.009			
TD-_LA_	1.57	1.17–2.20	0.005	0.045	0.36	0.15–0.87	0.024
VD-_LA_	1.74	1.22–2.59	0.004	0.036			
AD-_LA_	2.47	1.56–4.16	<0.001	0.009			
Volume	1.01	1.01–1.02	<0.001	0.009	1.02	1.01–1.04	0.002
Location	8.31	3.53–22.10	<0.001	0.009	7.90	2.64–23.65	<0.001
Shape	9.31	3.81–26.50	<0.001	0.009	11.80	4.01–34.72	<0.001

Abbreviations: See Table 2 and Table 3. for abbreviations of characteristics. OR, odds ratio; CI, confidence interval; Corrected-*p*, *p* value was corrected via Bonferroni correction.

**Table 5:** Optimal radiomics features and their respective coefficients.

Variables	Radiomics Feature Name	Coefficient
A	wavelet.HHL_glszm_ZoneEntropy	−0.2842
B	wavelet.LLL_glcm_IMC1	1.3021
C	wavelet.LLH_glszm_SmallAreaLowGrayLevelEmphasis	−0.4753
D	wavelet.LHL_firstorder_Median	0.5513
E	logarithm_glcm_inverseVariance	−1.1622

Abbreviations: glszm indicates gray-level size zone matrix; glcm, gray-level co-occurrence matrix; IMC1, informational measure of correlation 1.

**Table 6:** Performance of all four prediction models.

Group		AUC	Sensitivity	Specificity	Accuracy	Precision
			(%)	(%)	(%)	(%)
Rad-score	training set	0.82	70.8	80.6	75.7	78.5
		(95%CI: 0.75–0.89)				
	test set	0.82	76.7	63.3	70.0	67.6
		(95%CI: 0.72–0.93)				
Rad-clinic score	training set	0.86	72.2	80.6	76.4	78.8
		(95%CI: 0.80–0.92)				
	test set	0.82	80.0	66.7	73.3	70.6
		(95%CI: 0.71–0.93)				
Rad-radio score	training set	0.90	75.0	80.6	77.8	79.4
		(95%CI: 0.85–0.95)				
	test set	0.84	86.7	63.3	75.0	70.3
		(95%CI: 0.75–0.94)				
Nomo-score	training set	0.93	76.4	87.5	81.9	85.9
		(95%CI: 0.89–0.97)				
	test set	0.85	90.0	66.7	78.3	73.0
		(95%CI: 0.76–0.95)				

Abbreviations: Rad-score was calculated based on radiomics model; Rad-clinic score was calculated based on radiomics-clinical model; Rad-radio score was calculated based on radiomics-radiological model; Nomo-score was calculated based on combined model; AUC, area under the receiver operating characteristic curve; CI, confidence interval.

**Table 7:** The DeLong test results of the four models.

Group	Model A	Model B	AUC of Model A	AUC of Model B	*p*-Value
training set	Nomo-score	Rad-score	0.93	0.82	0.002
	Nomo-score	Rad-clinic score	0.93	0.86	0.006
	Nomo-score	Rad-radio score	0.93	0.90	0.204
	Rad-score	Rad-clinic score	0.82	0.86	0.989
	Rad-score	Rad-radio score	0.82	0.90	0.006
	Rad-clinic score	Rad-radio score	0.86	0.90	0.049
test set	Nomo-score	Rad-score	0.85	0.82	0.147
	Nomo-score	Rad-clinic score	0.85	0.82	0.079
	Nomo-score	Rad-radio score	0.85	0.84	0.743
	Rad-score	Rad-clinic score	0.82	0.82	0.710
	Rad-score	Rad-radio score	0.82	0.84	0.183
	Rad-clinic score	Rad-radio score	0.82	0.84	0.179

Abbreviations: AUC, area under the receiver operating characteristic curve.

## Data Availability

The data presented in this study are available on request from the corresponding author.
